# Annotations

**Published:** 1892-03-05

**Authors:** 


### Annotations.

**Opium.** The Anti-opiumists have published the names of some 5,000 medical men of the United Kingdom, who endcrse the opinions that the habit of opium smoking or of opium eating is morally and physically debasing; that the unrestrained sale of such a drug as opium is immediately associated with many and grave dangers to the well-being of the people of India; and that the drug ought in India, as in England, to be classed and sold as a poison, and purchased from chemists] only. They are, therefore, further of opinion that the Government of India should prohibit the growth of the poppy, and the manufacture and sale of opium, except as required for medical purposes. Now amongst the mass of contradictory writing as to the evil effects, or otherwise, of the use of opium, it is difficult to arrive at any definite conclusion, and we scarcely think that the opinions of the 5,000 medical men of the United Kingdom will carry much weight, for it is not possible that many of these practitioners can have had any practical experience of the evil effects of opium, among those using opium habitually. On the other hand, we have such authorities as Sir Lepel Griffen, Sir George Bridwood, Mr. Brereton, Dr. Ayres, Sir William Moore, Dr. Lisboa of Bombay, and many others, stating that the sensational accounts given of opium smokers and of opium dens, are drawn from exceptional instances. We certainly should be very pleased if it were possible to limit the use of opium to physicians' prescriptions, but we fear this cannot be accomplished, any more than the use of spirits or beer could be prohibited in this country. Lord Lansdowne, the Viceroy of India, has recently said that it is beyond our power to put an end to the use of opium in China and in India. The cultivation of the poppy could not, indeed, be prohibited in India, without infraction of our treaties with those native states in which opium is produced; for it would be interference with the internal affairs of the State. It should be recollected that opium is often used, not only in India, but also in England, as a preventive against malarious fever, and this could not be done if a physician's prescription was required every time opium was so used. There are certain benefits connected with the use of opium which, we think, are not sufficiently appreciated by the anti-opiumists. The Government of India are endeavouring to restrict the use of opium as much as possible, and this we opine is all that can be done. It should be recollected that those using opium rarely use fermented liquors. Some kind of stimulant always has been, and always will be desired and obtained by the masses. Driving the people of India or of China to the use of spirituous liquors, in place of opium, would be at the most, a doubtful benefit.

**Criminal Anthropology.** The Congress of Criminal Anthropology will hold its meeting at Brussels in August and September of the present year. The careful and dispassionate study of criminals from a purely scientific standpoint is one of those distinguishing circumstances which mark off modern from mediæval and ancient times. A rough-and-ready promptitude was the chief characteristic of the behaviour of all classes of persons towards known criminals in almost all centuries and countries before the dawn of modern science. But it is now recognised that the State and the public owe at least one duty, even to criminals, namely, that of taking the trouble to understand them. It is not too much to say that every human being is a potential criminal, exactly as he is a potential epileptic, or a potential lunatic. A single blow on Shakespeare's skull at the age of five-and-twenty might, by fracturing its inner table, and causing a certain amount of pressure upon the brain, have deprived us of "Hamlet" and "King Lear," and all the other plays which have made the name of Shakespeare a household word in every civilised country. An obstruction in one of the blood-vessels of Mr. Gladstone's head might have converted the bright and vivacious old statesman of the last ten years into a mere mumbling imbecility. The shape of a man's cranium can alter his character. The want of nourishing food can impair intelligence; a poisonous atmosphere may produce an irritable nervous system, and in the long run criminality or lunacy. These are facts, not fancies and fads. They are facts which science has not only recognised, but verified again and again. But if they be facts, they should be taken into account by all sorts of persons who have anything to do with criminals; by the parents of children who commit crime; by friends whose trusted companions disappoint and deceive them; by judges and juries who have control of the liberty and life of criminals. It must be admitted that justice is to be done even on behalf of those who commit crimes. Nay, the very act of crime so places a man in antagonism against all the rest of the world that the criminal becomes at once entitled to a certain amount of help and defence against odds that would otherwise be altogether too overwhelming. The laws of most civilised countries recognise this already, and in a mere spirit of fairness assign to every unaided criminal an advocate who can presumably put his case in the best possible light. What the law already does in an elementary kind of way, science, out of her mere love of truth and justice, strives to get done as completely as possible. And in order to bring about this advance in civilisation, she gathers facts from every quarter of the globe; she collates them; she considers and weighs them with careful accuracy, and when she has done all these things, she presents the facts to judges, clergymen, fathers of families, friends, employers of labour, and indeed to all sorts and conditions of men in order that they may become wiser, fairer, and more helpful persons to criminals as well as to the rest of the world.

**Unhappy Shop Assistants.** The *Lancet* has done well to start a crusade in favour of shop assistants, and against the unhealthy and miserable environment in which their daily work is carried on. As becomes a grave and reputable newspaper, the tone of the discussion is severely sober and free from exaggeration. But in the judgment of all sensible persons the statements of the *Lancet* will lose none of their weight on that account. The indictment drawn up against the proprietors and managers of town shops is of a very serious character, and demands the attention not only of shopkeepers themselves, but also of the public guardians of the interests of our industrial population. It appears that large numbers of shop assistants, both male and female, are ill-paid; are indeed paid much less than the wages of skilled mechanics. Not only are they ill-paid, but they are under-fed, abominably under-fed. As a sample of the under-feeding, we are told a story of a certain draper in a West end suburb. "This draper was stated to have usually gone to a butcher's between eleven and twelve o'clock on Saturday nights, just when the shop was about to close. He would then offer to purchase all the remnants, or 'block ornaments,' at 2d. a pound. As these fragments at so late an hour could not be sold, the butcher generally accepted the offer, and the draper, having arranged a method for keeping this meat in ice, fed his assistants during the ensuing week." One would like to know whether the draper himself and his family helped to consume these choice and dainty remnants. Another count in the indictment is that the sanitary condition of some of the eating and sleeping apartments provided for shop assistants is both disgusting and dangerous. "At one large grocery shop the assistants complained that they were made to take their meals in a dark basement below the level of the street. Here was situated an unventilated closet, with no light and no window. The odour of the drains pervaded the whole place. While some grocers allow their assistants to eat their meals near unventilated drains, others do not hesitate to store their provisions round and about closets." This is too much for our patience; we do not hesitate to say that grocers such as these richly deserve to be sent to prison. One count more is all we have space for. The mechanic as a rule works from forty-eight to fifty-four hours a week; a shop assistant, it is asserted, works from seventy to ninety hours a-week, that is, on an average, from twelve to fifteen hours a day. It is plain that, even in England, there is ample work for those who charge themselves with the care of the feebler and more defenceless members of society. It should be borne in mind that shop assistants are not like middle-aged and strong-bodied mechanics and labourers, who, if they leave one situation, can usually get another, without so much as being asked for a character. Those who toil in shops are either young women and girls, or comparatively young and inexperienced men. Their employers have them almost entirely in their power, in that a new situation can seldom be secured, except by means of a character from the old one. The philanthropic public will do well to insist that the establishments of retail traders shall be placed under Government inspection and control as fully and completely as factories and workshops are.

